# Cancer Biomarkers: Are We Ready for the Prime Time? 

**DOI:** 10.3390/cancers2010190

**Published:** 2010-03-22

**Authors:** Alok Mishra, Mukesh Verma

**Affiliations:** 1 Institute of Cytology and Preventive Oncology, Division of Molecular Oncology, Noida, 201301, UP, India; E-Mail: mishraa2@mail.nih.gov; 2 Methods and Technologies Branch, Epidemiology and Genetics Research Program, Division of Cancer Control and Population Sciences, National Cancer Institute, National Institues of Health (NIH), 6130 Executive Blvd., Suite 5100, Bethesda, MD 20892-7324, USA

**Keywords:** biomarker, cancer, diagnosis, glycans, prognosis, validation

## Abstract

A biomarker is a characteristic that is objectively measured and evaluated as an indicator of normal biologic processes, pathogenic processes, or pharmacologic responses to a therapeutic intervention. In cancer, a biomarker refers to a substance or process that is indicative of the presence of cancer in the body. A biomarker might be either a molecule secreted by a tumor or it can be a specific response of the body to the presence of cancer. Genetic, epigenetic, proteomic, glycomic, and imaging biomarkers can be used for cancer diagnosis, prognosis and epidemiology. These markers can be assayed in non-invasively collected biofluids. However, few cancer biomarkers are highly sensitive and specific for cancer detection at the present time. Consequently, biomarkers are not yet ready for routine use due to challenges in their clinical validation for early disease detection, diagnosis and monitoring to improve long-term survival of patients.

## 1. Introduction: Defining Biomarkers

According to the US National Institutes of Health’s (NIH) Working Group and the Biomarkers Consortium, a biomarker is a characteristic that is objectively measured as an indicator of normal biological processes, pathogenic processes, or a pharmacological response to a therapeutic intervention (http://www.biomarkersconsortium.org). The NIH’s National Cancer Institute (NCI) (http://www.cancer.gov/dictionary/?CdrID=45618), describes biomarkers in its dictionary of cancer terms as “A biological molecule found in blood, other body fluids, or tissues that is a sign of a normal or abnormal process, or of a condition or disease. A biomarker may be used to see how well the body responds to a treatment for a disease or condition. Biomarkers are also called molecular marker and signature molecules.”

Others define a biomarker as a measurable phenotypic parameter that characterizes an organism’s state of health or disease, or a response to a particular therapeutic intervention. Biomarkers can also be defined as physical, chemical, or biological agents accessible in body matrices that can be measured in body fluid or cells. The United Nations’ World Health Organization (WHO) defines a biomarker as any substance, structure or process that can be measured in the body or its products and influences or predicts the incidence of outcome or disease (Biomarkers in Risk Assessment: Validity and Validation, Environmental Health Criteria Series, No222, WHO). In the following sections we discuss different classifications of cancer biomarkers and their clinical implications and current challenges in the field.

## 2. Historical Perspective

Perhaps the earliest examples of cancer biomarkers are urinary Bence Jones protein and tumor specific antigen carcinoembryonic antigen (CEA) in colon carcinomas [1]. Lander’s group used genomic signatures as a biomarker for the classification of cancers [2]. However, the prostate specific antigen (PSA) has been the most important early use of human cancer biomarkers in a clinical setting [3,4]. PSA is a biomarker for prostate cancer and is still used in clinics today [5,6]. 

## 3. Classification of Cancer Biomarkers

**Figure 1:**
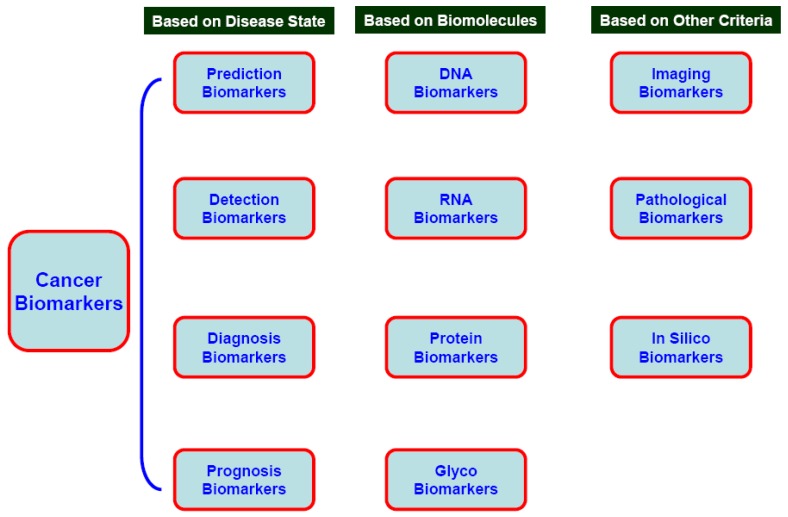
Classification of Biomarkers.

Several attempts have been made to define and classify cancer biomarkers using different approaches, but general consensus has yet to be established. Broadly, any biologically derived entity or processes which lead to a cancer diagnosis (in prognosis, screening and risk assessment), at the stage of diagnosis or post diagnosis (in therapy and treatment module) are potential candidates as cancer biomarkers [7,8,9,10,11,12,13,14,15,16,17,18,19]. Due to the vast explosion of knowledge over the past several decades, collectively and in multiple spheres of the biomedical sciences and technology development, different methods have been suggested to classify cancer biomarkers. But these classifications should be considered contextual as identification of cancer biomarkers is one of the major multidisciplinary areas of the biomedical field. A schematic for the classification of biomarkers is shown in Figure 1. The following section is an attempt to classify cancer biomarkers according to contemporary findings. It is important to note that some of the biomarkers in the following categories are overlapping in nature, *i.e.*, biomarkers for cancer screening and prediction might also be useful for cancer grading or staging [8,19].

### 3.1. Prediction, Detection, Diagnostic, Prognostic, and Pharmacodynamics Cancer Biomarkers

Prognostic biomarkers are based on the distinguishing features between benign and malignant tumors. These biomarkers may also be chosen based on the differentiation status of tumors which can influence clinicians’ decisions related to treatment modalities. For example, the prognosis for human papillomavirus (HPV)-associated oral tumors is relatively good in terms of survival time because they manifest in a comparatively well differentiated state [20]. Such markers are also important from the point of view of predicting relapse of oral cancer. Commercially available tests such as Oncotype DX (Genomic Health), Mamma Print (Agendia) and the H/I (AviaraDx) are popular in deciding the clinical outcome after surgery on the basis of genetic expression readout.

Predictive biomarkers, sometimes referred to as response markers, are utilized exclusively in assessing the effect of administering a specific drug. These biomarkers allow clinicians to select a set of chemotherapeutic agents which will work best for an individual patient. For example, Herceptin® is useful in breast cancer lesions showing only *Her2/Neu* overexpression, whereas tamoxifen is the preferred treatment for other breast cancer lesions. Thus *Her-2/Neu* is a predictive cancer biomarker for a subset of breast cancer therapies [21]. Likewise, drugs such as erlotinib or gefitinib work only in lung cancer patients with specific mutations in the epidermal growth factor receptor (EGFR) gene [22]. Another cited example is the use of Gleevec®, restricted to certain types of leukemia with Philadelphia chromosome [23]. Gleevec targets one cancer protein that causes Philadelphia chromosome positive chronic myeloid leukemia and another protein, Kit, which is the suspected cause of gastrointestinal stromal tumors.

Pharmacodynamic markers are cancer markers which are utilized in selecting doses of chemotherapeutic agents in a given set of tumor-patient conditions. These markers help in optimizing cancer drug doses below their cytoxicity level and phasing the clinical trials to next level.

Diagnostic markers may be present in any stage during cancer development [14,24]. Calcitonin in medullary thyroid cancer (MTC) is an example of a diagnostic marker present in the early stages of cancer. Moreover, a diagnostic cancer marker can be stage, tissue, relapse, follow-up and age specific. HPV is considered to be a diagnostic cancer biomarker for uterine and cervical cancers as it is present in >90% cancer lesions. The use of HPV as a diagnostic biomarker has been a major step in the development of a cervical cancer screening program and in vaccine development. Recently, the US Food and Drug Administration (FDA) approved some diagnostic markers for bladder cancers based on urine analysis, such as bladder tumor antigen (BTA) and nuclear matrix protein-22 (NMP-22) [25]. Survivin and calreticulin also have diagnostic potential for bladder cancer [26,27].

### 3.2. Cancer Biomarkers on the Basis of Biomolecules

#### 3.2.1. DNA

Single nucleotide polymorphisms (SNP) in many genes are major DNA markers, including *XRCC1*, *ATM*, *p53* (lung, head, and neck cancers); *CYP1A1*, *RAD1*, *BRCA1* and *BRCA2* (breast cancer); and *PGS2* (lung cancer). Other major DNA markers include loss of hetrozygosity (LOH); variation in copy number of genes; chromosomal aberrations at a gross cytogenetic level, such as translocation/fusion (BCR-ABL, PML-RARA translocation in leukemias), micro-satellite instability (MSI), and epigenetic modifications [7,14,19,28]. Mutation(s) in DNA nucleotides in tumor promoters (*Ras*, *APC*), tumor suppressors (*p16*, *p53*, *p19*, *Rb*), cell cycles (cyclins), and DNA-repair related genes (*XRCC*) have been associated with prognosis and diagnosis of different cancers, although their clinical implications have yet to be established. The source of DNA may be from tissue, serum, sputum, saliva, bronchial tear, cerebrospinal fluid (CSF), and tumor cells circulating in the blood, bone marrow, and nipple aspirate [18,28,29]. Interestingly, besides nuclear aberrations, alterations in mitochondrial DNA (mtDNA) molecules are suggested strongly as biomarkers for numerous cancers [15,18,30,31].

Epigenetic modification of nucleic acids and associated proteins (histones and non-histones) are important in carcinogenesis [19,32,33]. Histone deacetylation, lysine-specific histone-H3 methylation, and promoter region CpG methylation modulates transcription of tumor-suppressor genes (*CDKN2A*, *TP53*, *APC*, *BRCA1*); DNA mismatch-repair genes (*MLH1* or the *O6-methyl-guanine-DNA methyltransferase gene*, *MGMT*). Gene silencing by CpG methylation is one of best characterized epigenetic modifications to date [19,30,33]. The degree of methylation in prostate cancer tissue, sputum/serum from patients with lung cancer, and saliva in those with oral malignancies are directly implicated in the severity of the lesions. Repetitive DNA sequences, such as those belonging to the *Alu* family, are generally found in regions of DNA termed “satellite” DNA and are associated primarily with the pericentric (next to the centromere and at the centromere/ juxtacentromeric and centromeric) heterochromatic region of metaphase chromosomes. In cells of normal postnatal somatic tissue, repetitive sequences are relatively enriched in 5-methyl cytosine (m^5^C) compared to the genome as a whole. However, in sperm cells, the normal methylation pattern of these repetitive regions of DNA is lower than that seen in most somatic cells. In virtually any other context, hypomethylation of repetitive sequences is generally indicative of malignancy. For example, hypomethylation of satellite DNA has been observed in ovarian tumors, and the degree of hypomethylation correlates with the malignant potential of the tumor based on histological criteria. 

#### 3.2.2. RNA and Micro RNA (miRNA)

Some of methods used to detect cancer biomarkers at the RNA expression level include Quantitative Reverse Transcription Polymerase Chain Reaction (RT-qPCR), Serial Analysis of Gene Expression (SAGE), differential display, bead-based methods, and microfluid card and micro-array analysis [34]. Pure RNA signature procurements are attempted by laser capture-based microscopy in different grades and stages of therapy. Comparative analysis of RNA expression in terms of heat maps, supervised-algorithms, and snapshots are eventually linked with diagnosis and prognosis [34,35,36,37,38]. 

Micro RNAs (miRNAs) are small non-coding RNAs. The expression of specific populations of miRNA in a tissue- and time-dependent manner is associated with clinical characteristics for several cancer types, such as leukemia, breast, prostate, colorectal, hepatic, lung, and pancreatic cancers [39,40,41]. There is sufficient evidence indicating that miRNA expression profiles can be used to classify human cancers, which also suggests a correlation exists between disease prognosis and therapeutic outcome. The area of metastasis-associated miRNA markers in relation to oncogenesis is expanding rapidly and these markers have recently been referred to as “metastamirs” [42]. miRNA can act as a tumor suppressor as well as an oncogene [43]. For example, miR15a is a suppressor for *Bcl-2* in chronic lymphocytic leukemia (CLL), prostate cancer, and myeloma. let-7 is a suppressor for *RAS* in lung and gastric cancers and mir17 and mir21 clusters modulate *PTEN*, *TGFβ-RII* and are oncogenic for many lymphomas; blastomas; and prostate, breast, and lung cancers. These observations emphasize the potential application of miRNAs as biomarkers for diagnosis, prognosis, stage, risk stratification and prediction, and drug- responses in patients with cancer.

#### 3.2.3. Protein Markers

Because proteins are the main executioner bio-molecules in cells, protein-based markers are more important biomarkers than DNA- or RNA-based markers [44,45]. Protein molecules influence the molecular pathways in normal and transformed cells; therefore, proteomic markers are closer and more relevant to the disease state initiation and progression. The only FDA-approved biomarkers currently available for clinical use are protein molecules. Protein-based signatures are derived from the techniques of classical two-dimensional (2-D) fluorescence difference gel electrophoresis (DIGE); polycarylamide gel electrophoresis (PAGE); and high throughput platforms, such as Mass Spectroscopy (MS), Matrix Associated Laser Absorption Desorption Ionization Time of Flight (MALDI-TOF), Surface Enhanced Laser Absorption Desorption Ionization Time of Flight (SELDI-TOF), and reverse phase microarray [31,45,46,47,48]. Quantum dots and nanoparticles are recent additions to the technologies available to assess the potential of protein molecules as cancer biomarkers [49]. Quantitative proteomics have been utilized to discover cancer biomarkers in different organ sites, such as Stable Isotope Labeling with Amino Acids in Cell culture (SILAC) for prostate cancer [46]; iTRAQ for leukemia [50]; Liquid Chromatography-Mass Spectrometry/Mass Spectrometry (LC-MS/MS); antibody arrays [51]; bead suspension arrays for cervical and ovarian cancers; and aptamer arrays for breast, lung and colorectal cancers [52,53]. 

#### 3.2.4. Carbohydrate Biomarkers

During the progression of some cancers, the expression of certain N-linked and O-linked glycans changes. These altered glycoforms can serve as candidate biomarkers for cancer detection [54,55,56]. Mass spectrometry is generally used to detect disease-associated carbohydrate markers. Tissue samples and biofluids (serum, cerebrospinal fluid, pancreatic fluid, lavage) are suitable for detecting breast, colon, ovarian, pancreatic, lung and colon cancers [57,58]. Serum glycomics have been utilized recently to detect esophageal cancer [59].

Since glycomarkers (glycoproteins, proteoglycans and glycolipids) are more stable than RNA and proteins, these markers are more suitable for epidemiological studies where human populations can be screened to identify those who are likely to develop cancer in their lifetime. Profiling O- and N-linked glycosylation of protein molecules at serine and threonine residues by MALDI-TOF and Electrospray Ionization (ESI) in human sera, tissue and cancer lines are an important approach to detect glycan-based cancer biomarkers. Increased branching and altered terminal structures of glycans are due to modulated expression in glysyltransferases (sialyl and fucosyl-transferases). Some of the most common terminal glycan moieties found in cancer cells are sialyl Lewis x (sLex), sialyl Tn (sTn), Globo H, Lewis y (Ley) and polysialic acid, as described in the literature [60]. Many O-linked glycans are not present in ovarian cancer patients’ serum, therefore, it is important to note that neoexpression of glycans as well as altered expression can serve as potential cancer biomarkers [61]. 

### 3.3. Pathogenic Cancer Markers

#### 3.3.1. Viral markers

Infectious agents in general and viral infection in particular contribute to ~15–20% of all human cancers, consequently, the presence of viruses with specific tumor types makes viruses highly attractive biomarkers [44,62]. The presence of Epstein–Barr Virus (EBV) is linked with nasopharyngeal carcinoma and lymphoma. HPV is associated with cervical cancers and subsets of head and neck cancers. Viral hepatitis B (HBV) and hepatitis C (HCV) have been associated with hepatocellular carcinoma, which is significant because liver cancer is the third most common cause of cancer-related death worldwide [63]. Hepatitis E infection is endemic to developing countries and it has been predicted that plasma transthyretin and urine alpha1m could be reliable biomarkers of acute hepatitis E infection [63]. Other viruses which have been associated with cancer include Kaposi’s sarcoma associated herpesvirus and human herpesvirus 8 (KSHV/HHV-8) which are associated with sarcoma and lymphoma and RNA viruses such as human T-cell lymphotropic virus type 1 (HTLV-1) are an etiological factor for certain types of leukemia [64]. 

#### 3.3.2. Bacterial Markers

*Helicobacter pylori* (*H. pylori*) cause a chronic low-level inflammation of the stomach lining. *H. pylori* infection is strongly linked to the development of duodenal and gastric ulcers and is an established biomarker for gastric cancer [65,66]. More than 50% of the world's population harbor *H. pylori* in their upper gastrointestinal tract. Infection is more prevalent in developing countries. Over 80% of individuals infected with the bacterium are asymptomatic. Either DNA polymorphisms or antibody-based technologies are used to detect *H. pylori* in patients. Antibiotics are effective against this bacterium and eradication of the infection in individuals will improve symptoms including dyspepsia, gastritis and peptic ulcers, and may prevent gastric cancer.

#### 3.3.3. Imaging Markers

Physical examinations and non-invasive technologies are not always sufficient for early detection of cancer. Current imaging techniques, such as x-ray, computed tomography (CT), ultrasound, radionuclide imaging, and Magnetic Resonance Imaging (MRI), have been used widely for cancer screening and diagnosis, including disease staging, as well as determining the efficacy of cancer therapy and monitoring disease recurrence [67]. In prostate cancer, attempts have been made to correlate PSA expression with bioimaging data [68]. Mammograms are extensively used for screening women over age 50 to detect breast cancer. According to a recent report from American Cancer Society (ACS), the rate of breast cancer has declined due to screening practice. Colonoscopy is routinely done for screening populations at high risk of developing colon cancer.

## 4. Bioinformatics and Cancer Biomarkers

Cancer subtypes and biomarkers have been identified using technologies that combine clustering algorithms and visualization tools into a Web-based application and those that analyze high-throughput gene expression data using various case-control models [69]. Popular analytical tools include the following: MAGMA (www.magma-fgcz.uzh.ch) for statistical analysis; Interwoven Loop or ILOOP for designing arrays; Gene Expression Profile Analysis Suite or GEPAS (http://www.gepas.org) and CARMAweb (https://carmaweb.genome.tugraz.at) for microarray analysis; GenePattern (https://cabig.nci.nih.gov/tools/GenePattern#download) for expression data analysis; GoMiner (http://discover.nci.nih.gov/gominer), GOStat (http://gostat.wehi.edu.au), AmiGO (http://amigo.geneontology.org/cgi-bin/amigo/go.cgi), BiNGO (http://www.psb.ugent.be/cbd/papers/BiNGO/), and GOEAST (http://omicslab.genetics.ac.cn/GOEAST/) for gene ontology analysis. These tools are used as *in silico* or bioinformatics tools in the process of cancer biomarker discovery. RMA Express (http://rmaexpress.bmbolstad.com/RMAExpress), dChip (http://www.dchip.org/automate.htm), and caCORRECT (http://cacorrect.bme.gatech.edu/) are used during normalization, quality control, and interpretation of expression array data. In general, Gene Expression Omnibus (http://www.ncbi.nlm.nih.gov/geo/), ArrayExpress (http://www.ebi.ac.uk/microarray-as/ae/), caArray (https://cabig.nci.nih.gov/tools/caArray), ArrayWiki (http://arraywiki.bme.gatech.edu) are used for storage, dissipation, and management of expression data by bioinformaticians. OmniBiomarker (http://omnibiomarker.bme.gatech.edu/) is a Web-based bioinformatics tool for developing biomarkers in oncology which has many small modules to perform initial step such as selection of samples to predict the final clinical outcome of potential biomolecules as a biomarker. This tool is used extensively in the discovery of renal cell carcinoma biomarkers; The NCI’s Cancer Biomedical Informatics Grid® (caBIG®) initiative and its tools are among the most widely used tools at each stage of cancer biomarker discovery from selection of target groups to clinical trials and validation of molecules by clinical scientists. At the same time, caBIG® also supplies information related to basic research to share free of charge.

## 5. Cancer Biomarkers for Selected Organ Sites

### 5.1. Lung

Lung cancer is the leading cause of cancer-related mortality in the world. The most commonly utilized serum markers of lung cancer include squamous cell carcinoma antigen, carcino emryonic antigen (CEA), neuron-specific enolase (NSE), CA-125 [70], CYFR A 21–21 (cytokeratin fragment 21], chromogranin A, retinol-binding protein (RBP), and α1-antitrypsin. Overactivation of oncogenes, such as *K-ras* [71], myc, *EGFR* [72] and *Met* [73], or inactivation of tumor suppressor genes, such as *p53* [74] and *Rb* [75] are other biomarkers for lung cancers. Some reports also suggest that *TTF-1*, *Pax9*, and *Nkx-8* amplification at the DNA level plays a role in lung cancers. Additionally, hypermethylation of *p16*, *RARB*, and *DAPK* genes may predict development of lung cancer [76].

### 5.2. Uterine and Cervical Cancers

HPV infection and oncogene E6 and E7 expression are the most important markers implicated for uterine and cervical cancer in women [77]. Overexpression of mini chromosome maintenance (MCM) proteins is seen in severe dysplastic lesions [78]. Similarly, overexpressed cell division cycle protein 6 (CDC6) is observed in malignant cervical cancer [79]. p16^INK4A^ is a marker of squamous and glandular dysplastic cervical epithelium [80]. The ribosomal protein S12 gene has also been reported as an early molecular diagnostic identifier for the screening of human cervical cancer and is a potential target in cancer gene therapy trials [81,82]. Recently, SWI/SNF (SWItch/Sucrose NonFermentable) stabilizing molecule SMARCC1 has been detected in early dysplastic stage and has potential as a predictive marker. Upregulated hTR and hTERT subunits of telomerase have also been observed in cervical cancers [83].

### 5.3. Breast Cancers

The American Society for Clinical Oncology (ASCO) recommended eight different protein-related tumor markers for breast cancer: CA 15–13, CA 27–29, carcinoembryonic antigen, estrogen receptor (ER), progesterone receptor, human epidermal growth factor receptor 2 (HER2), urokinase plasminogen activator (uPA), and plasminogen activator inhibitor (PAI)-1. CA 15–13, CA 27–29 and carcinoembryonic antigen are biomarkers for monitoring; estrogen receptor (ER), progesterone receptor (PR), and HER2 are markers for treatment planning; and uPA and PAI-1 are biomarkers for recurrence risk prediction [84]. Other potential markers include p53, cathepsin D, cyclin E, and kallikrein 14 [84,85]. MapQuant Dx™ Genomic Grade platform is based on the mRNA expression of about 100 genes for breast cancer detection and BCtect™ is a RT-PCR-based assay with several genes for early detection. Studies have shown that miRNA markers (mir-125b, mir-145, mir-21 and mir-155) are dysreguled in breast cancers [86]. Gene-expression signatures in breast cancer have been extensively reviewed [87], which led to the classification of treatment modalities for breast cancer patients on the basis of mRNA transcript expression patterns. Genes coding for *cyclin D2*, and *RAR-β* are epigenetic markers. Hypermethylation of one or more genes (*BRCA1*, *p16*, *and 14-3-3* *σ*) was found in 95% of sporadic breast cancers [88]. Serum HER2 has emerged as a biomarker candidate while cytokeratins 8, 18, and 19 are proposed cancer makers. Kallikrein, osteopontin, mutp53 and crypto1 are additional cancer biomarkers for breast cancers. Nanotechnology approaches are being developed to identify markers in breast cancer [89].

### 5.4. Liver Cancer

Alphafaetoprotein [(AFP), AFLP (*Lens culinaris* –a derivative for AFLP)] and DCP (des- carboxy prothrombin) are the most utilized popular cancer biomarkers for liver cancer or hepatocellular carcinoma (HCC) known so far [90,91,92]. Transforming growth factor beta 1 (TGF-beta-1), I-6/10, IGF, and gamma glutamyl transferase (GGT) enzyme levels are proposed cancer biomarkers [93,94]. Other less established markers are the expression level of Glypican-3 (GPC3) and Golgi Protein 73 (GP73). 

### 5.5. Prostate Cancer

Although PSA is a popular clinical biomarker for prostate cancer, data from the American College of Surgeons’ National Cancer Data Base (NCDB), indicates that it is not associated with cancer in every patient. Therefore, many other potential cancer biomarkers for prostate cancer are being investigated. Different subtypes of PSA are known and percent of subtypes of PSA, fPSA, is also important to know the aggressiveness of lesion along with another subtype, tPSA [95]. Many investigators have reported other potential molecular markers for this cancer, including the following: overexpression of human kallikrein-related peptidase 2 (hK2), early prostate cancer antigen (EPCA), α-methylacyl-coA racemase (AMACR), insulin-like growth factors and binding proteins (IGFBP-2 and IGFBP-3), TGF-β1, elevated circulating levels of the cytokine interleukin-6 (IL-6) and its receptors, urokinase plasminogen activator (uPA) and receptor (uPAR), enhancer of zeste homolog 2 (EZH2), and prostate-specific membrane antigen (PSMA) [96,97,98,99,100,101,102,103].

### 5.6. Head and Neck Cancers

Mutations in the tumor suppressor *p53* gene have been observed in saliva and surgical margin analysis in head and neck squamous cell carcinoma (HNSCC) [104]. Other observed markers in head and neck cancers are LOH /microsatellite instability at 3p, 9p, 17p and 18q chromosomal locations, along with a hypermethylated promoter region of the *p16* gene [21,105]. Due to a lack of potential candidate biomarkers for HNSCC patients analogous to the ER or HER2 markers in breast cancers, or c-KIT in gastrointestinal cancers, genomic profiling studies may be useful for identifying new biomarkers with prognostic or predictive value. Aberrant mRNA transcripts of *EGFR*, *cytokerin 14 and 15*, *TGF-α*, *STAT-1*, *HLA-C*, and *GST-2* have been reported in HNSCC samples. Expression patterns of E18Ag, *Pemphigus vulgaris* antigen (PVA), and many cytokeratins have been implicated in the metastatic gradation of HNSCC [106,107]. Overexpressed telomerase, matrix metallopeptidase-9 and -2 (MMP-9 and MMP-2) [108] proteins are also biomarkers for HNSCC, along with nuclear factor- kappa beta (NF-κB) [109].

## 6. Concluding Remarks

### 6.1. Challenges in the Field and Potential Solutions

The number of biomarkers which are currently used in clinical settings is small because (i) preparation and storage of sample is not uniform; (ii) analytical validation of equipments used has not been accomplished; (iii) few techniques, such as mass spectrometry, provide huge amount of data which has not been analyzed efficiently; (iv) cancer samples have heterogeneity and most of the samples have not been collected and analyzed by laser capture microdissection; and (v) proper validation of most of the cancer biomarkers has not yet been achieved, although policies and guidelines to validate biomarkers have been developed (http://www.cancer.gov/cancertopics/factsheet/Detection/tumor-markers). ASCO, a nonprofit organization that represents more than 25,000 cancer professionals worldwide, has published clinical practice guidelines (http://www.cancer.gov/dictionary/db_alpha.aspx?expand=c#clinical%20practice%20guidelines) on a variety of topics, including tumor markers for breast and colorectal cancer (http://www.cancer.gov/dictionary/db_alpha.aspx?expand=c#colorectal%20cancer). The National Comprehensive Cancer Network^®^ (NCCN), another nonprofit organization, is an alliance of cancer centers, and they also provide Patient Guidelines, which include tumor marker information for several types of cancers (http://www.nccn.org/patients/patient_gls.asp). 

To overcome problems in cancer biomarker field and their implication in clinic a number of efforts are underway and we have discussed those in the following section. At the NCI, attempts are being made to identify and validate cancer biomarkers through a group of investigators who participate in the Early Detection Research Network (EDRN) (http://edrn.nci.nih.gov/). It is a consortium of over 300 investigators from 40 private or academic institutions that represents divergent scientific disciplines including genomics, informatics, proteomics, and public health. Other Federal collaborators include additional NCI programs, the National Institute of Science and Technology (NIST), the Centers for Disease Control and Prevention (CDC), the Jet Propulsion Laboratory (JPL), and the FDA. The EDRN is at a junction of taking the discoveries made and determining if there are appropriate clinical applications for them. Discoveries lead to work that confirms and improves the accuracy of the biomarkers, which then moves quickly to early clinical validation. Before the EDRN was formed, each part of this process was separate from the next step, and this slowed scientific progress. Through the EDRN, investigator-initiated projects are combined with a strong administrative and data infrastructure that requires and supports information sharing and collaboration among individuals and organizations. A pathway or process to test biomarkers in human biospecimens and bringing the information to clinicians has also been developed [110]. 

Five stages of biomarker validation have been defined by Pepe *et al.* [111]. For clinical implications, a biomarker needs to be validated in different institutes and in a large number of samples followed by approval from the FDA. The financial support can be arranged by public and private resources. Thus, the collaboration among investigators in universities and institutes, clinicians, industrial participants and FDA is a must to bring a biomarker from the lab to clinic.

In the methylation field (epigenetic markers), approaches such as direct sequencing and real-time quantitative PCR are being standardized and implicated so that methylation levels on both strands can be determined. Attempts are also being made to do methylation profiling, histone modifications, and miRNA profiling in same samples so that there is minimal variation due to samples. Furthermore, inclusion of multiple markers may increase the sensitivity and specificity of markers in diagnosing the disease. Factors that impact sensitivity and specificity include choice of clinicl specimen, e.g, urine or serum; specimen stability/degradation; processing of specimen, e.g., urine pellet (sediment) or supernatant; choice of target gene/primers; choice of technology for analysis; negative and no template control use to see whether reaction worked; efficacy of bisulfate treatment; and use of different areas of promoter of a gene for analysis. In proteomic markers, isotope coded affinity tag (ICAT) based markers have not been explored extensively in clinical samples.

An example of a recent successful clinical trial to validate different markers in colon cancer used either tissue or formalin fixed paraffin-embedded tissues [112]. Samples from more than 1,500 patients contributed by investigators from 31 countries were used in this prospective trial. Genotyping, loss of heterozygosity, and gene expression profiling were followed to validate the colon cancer markers. Tumor markers can be used in the detection, diagnosis, and management of some cancers. Although an abnormal tumor marker level may suggest cancer, this alone is usually not enough to diagnose cancer. Therefore, measurements of tumor markers are usually combined with biopsy results to diagnose cancer. Furthermore, patient related information (family history, diet and life style, behavior) helps tremendously in the accurate diagnosis of cancer. 

In closing, biomarkers offer great potential for improving management of cancer at every point from screening and detection, diagnosis, staging, prognosis, and assessment of treatment response.
